# Cyclooxygenase (COX) Inhibitors and the Newborn Kidney

**DOI:** 10.3390/ph5111160

**Published:** 2012-10-25

**Authors:** Francine G. Smith, Andrew W. Wade, Megan L. Lewis, Wei Qi

**Affiliations:** 1 Department of Physiology and Pharmacology, University of Calgary, Alberta, T2N 4N1, Canada; 2 Alberta Children’s Hospital Research Institute for Child and Maternal Health, University of Calgary, Alberta, T2N 4N1, Canada; 3 Department of Pediatrics, University of Calgary, Alberta, T2N 4N1, Canada

**Keywords:** perinatal, prostaglandins, newborn, patent ductus arteriosis, premature labour, indomethacin, COX-1, COX-2, neonate, COX inhibitors

## Abstract

This review summarizes our current understanding of the role of cyclo-oxygenase inhibitors (COXI) in influencing the structural development as well as the function of the developing kidney. COXI administered either during pregnancy or after birth can influence kidney development including nephronogenesis, and can decrease renal perfusion and ultrafiltration potentially leading to acute kidney injury in the newborn period. To date, which COX isoform (COX-1 or COX-2) plays a more important role in during fetal development and influences kidney function early in life is not known, though evidence points to a predominant role for COX-2. Clinical implications of the use of COXI in pregnancy and in the newborn infant are also evaluated herein, with specific reference to the potential effects of COXI on nephronogenesis as well as newborn kidney function.

## 1. Introduction

Cyclo-oxygenase (COX) is a rate limiting enzyme in the generation of the important family known as prostaglandins (PGs). Present in all tissues in the body, PGs regulate numerous physiological processes including gastric motility, platelet-mediated hemostasis, regulation of blood flow, inflammation, and kidney function [1,2,3]. Together, PGs comprise a diverse family of biologically active lipids derived from the enzymatic conversion of arachidonic acid by COX to PGG_2_/H_2 _followed by the generation of five primary bioactive prostanoids PGE_2_, PGI_2_, PGD_2_, PGF_2α_ and thromboxane A_2 _[4,5,6]. Although PG synthesis occurs in all cells and tissues, the kidney is a particularly rich source, with PGE_2_ being the major prostanoid excreted in the urine. Among the COX end-products, PGE_2_ is released in greatest abundance from all nephron segments [7] both basally and when stimulated, and can also be released, along with PGI_2_, from vascular endothelial and smooth muscle cells [8]. PGE_2_ interacts with four G protein-coupled E-prostanoid receptors, designated EP_1__–4_. Through these receptors, PGE_2_ influences a variety of physiologic functions in the mammalian kidney including renal vascular resistance as well as glomerular ultrafiltration, and tubular Na^+ ^transport [4,9].

Because COX enzymes are the rate-limiting step in the aforementioned cascade, the generation of PGs can be inhibited by the administration of COX inhibitors (COXI) collectively known as non- steroidal anti-inflammatory drugs (NSAIDs) [10]. It is the role of COXI on the development and function of the immature kidney that is the focus of this review.

## 2. COX Isoforms

Two main COX isoforms have been identified constitutive COX-1 and inducible COX-2, which catalyze the first step in the conversion of arachidonic acid to PGE_2_, I_2_, D_2_, F_2α_, and thromboxane A_2 _(TXA_2_) by the action of tissue-specific synthases and isomerases. The constitutively active COX-1 isoform is present in most tissues and is the predominant isoform, known to perform cellular housekeeping functions for regulation of physiological processes, whereas the levels of the COX-2 isoform are typically low and upregulated during inflammation. All major organs, such as the brain, heart, lungs, uterus and kidney, abundantly express both COX isoforms. The most recently discovered isoform, COX-3, is believed to be a splice variant of COX-1 [11], and also known as COX-1b [12]. Physiological functions of this isoform have not been characterized and will not be further discussed within this review.

**Figure 1:**
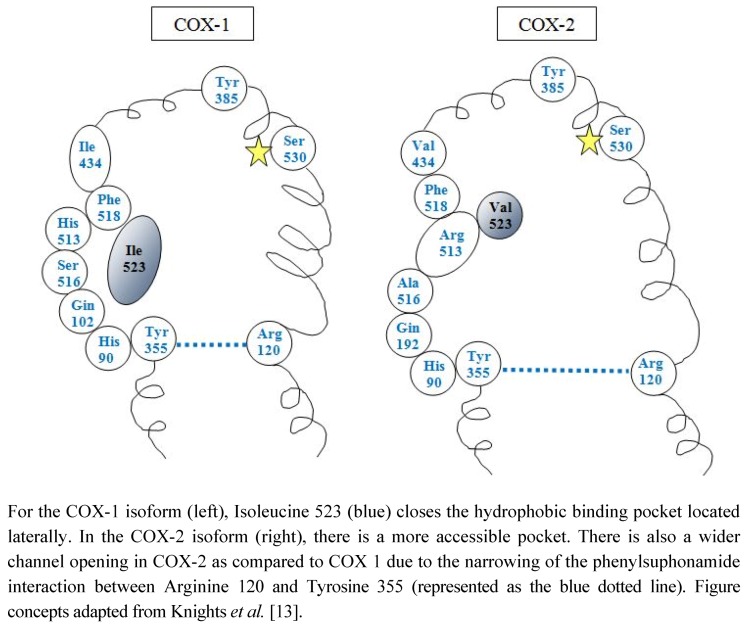
Structure of COX-1 and COX-2 isoforms.

Differences in the two predominant isoforms (COX-1 and COX-2) can be appreciated from their unique structures, as illustrated in Figure 1. Containing 576 and 587 amino acids, respectively, COX-1 and COX-2, also share 60%–65% sequence identities. Both enzymes are homodimers which each monomer containing three domains: (a) the N-terminal which holds the monomers together through hydrophic interactions, hydrogen bonding, and salt bridges, (b) the membrane binding domain, and (c) the C-terminus catalytic domain which comprises ~80% of the protein (or 480 amino acids) and contains both the COX and peroxidase active sites (Figure 1) [13]. COX-2 closely resembles COX-1 except that its active site accommodates larger chemical structures due to the substitution of isoleucine for valine at position 523 (shown as the blue amino acid in Figure 1). The associated loss of a methyl group with this substitution opens a secondary internal hydrophobic bonding site that increases the volume of the active site by ~25%. There are also smaller subtle changes that lead to a wider channel opening of ~20% in COX-2. For example, the phenylsuphonamide moiety between Arg 120 and Tyr 355 (shown as a blue dotted line in Figure 1) narrows the channel opening of COX-1 with respect to COX-2 (Figure 1).

### 2.1. COX Distribution in the Adult Kidney

**Figure 2:**
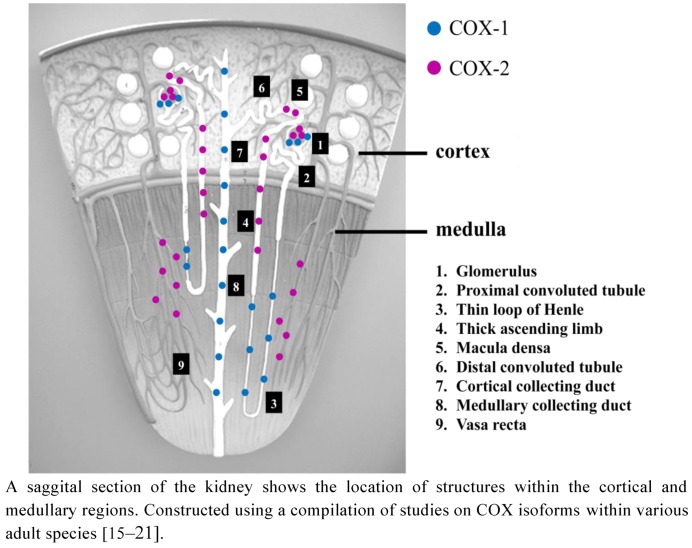
Distribution of COX isoforms in the adult kidney.

In the adult kidney, both COX-1 and COX-2 are present and localized to various structures implicating their roles in regulating renal haemodynamics and function (see Figure 2). COX-1 has been identified in arteriolar endothelial cells as well as mesangial cells of the glomerular tuft in adult bovine, ovine, rabbit, guinea pig, and rat [14]. In numerous animal species including bovine, ovine, simian, canine, and rodents, as well as in adult humans, COX-1 has been measured in cortical and medullary collecting tubules, interstitial cells and loop of Henle as well as parts of the renal vasculature [15]. COX-2 has been localized to podocytes and arteriolar smooth muscle cells, *macula densa* and the surrounding cortical thick ascending limb cells of the Loop of Henle, medullary interstitial cells as well as medullary *vasa recta* [16,17,18,19,20] (Figure 2). Their differential distribution implicates the two isoforms of COX as being involved in regulating different physiological functions within the kidney [21].

### 2.2. COX Distribution in the Developing Kidney

Several studies conducted in newborn animals have identified COX isoforms in the developing kidney. For example, in newborn rats, Stubbe *et al.* showed that COX-1 expression in the renal cortex remains constant throughout development whereas in the renal medulla, COX-1 expression increases eightfold from the first to fourth postnatal week [22]. For COX-2 expression, an increase in the cortex during week two to three of postnatal development in newborn rats was reported [22]. At the time of peak COX-2 expression at postnatal day 14, COX-2 mRNA levels were ten times higher in the cortex than the medulla. Zhang *et al.* showed that COX-2 expression in the rat kidney begins on day 20 in the *macula densa* with no expression found during the period of organogenesis (embryonic days 7 to 13). There is, however, diffuse cytoplasmic staining within cells of both the branching collecting ducts (ureteric buds) and the S-shaped bodies (mesenchyme) from embryonic day 16 [23]. At embryonic day 20, foci of intensely COX-2 positive tubular epithelial cells are found within the thick ascending limb, adjacent to *macula densa* cells. This expression is first noted in the juxtamedullary nephrons, but with centrifugal development of nephrons, there is movement of COX-2 positive cells outwards towards the cortex. After vascularization of the glomerulus, COX-2 positive cells first appear at the future site of the *macula densa* and then increase in number for the next seven to ten days in the developing rat kidney within the thick ascending limb of the loop of Henle [23].

In human fetal kidney, both COX-1 and COX-2 isoforms are also expressed. There is a relative abundance throughout gestation of COX-1 whereas COX-2 levels increase throughout gestation providing evidence for their roles in kidney development or function early in life: Kömhoff *et al.* studied nephrectomy specimens from seven adult human kidneys as well as tissue from fetal kidneys at 17 to 24 weeks of gestation [17]. In the human fetus, immunoreactive COX-2 was primarily expressed in podocytes, with increased expression extending from the early, comma-shaped, to the later vascularized, glomeruli [17]. Later in development, COX-2 was expressed mainly in endothelial and smooth muscle cells of both arteries and veins but also in the podocytes of juxtamedullary glomeruli. In contrast to that seen in adult rat and dog kidney [17,24], Kömhoff *et al.* reported that COX-2 is not expressed in *macula densa* cells in the developing human kidney [17]. Different results were, however, obtained more recently in another study of COX-2 expression in human fetal kidneys (15–23 weeks) by Khan *et al* [25]. In this study, COX-2 was shown to be strongly expressed in the *macula densa* of 19/23 fetuses and less strongly in occasional podocytes [25]. There was a progressive decline in COX-2 expression from the *macula densa* of subcapsullar glomeruli to that of more mature nephrons within the inner cortex [25]. Differences in these two investigations can be explained by differences in the techniques used in storing tissue for later evaluation, as well as the specificity of the antibodies used in the immunohistochemical analyses, with the latter study using anti-human COX-2 antibodies, whereas in the earlier study the antibodies may not have been specific for human COX-2.

## 3. The Role of COX Isoforms in the Developing Kidney

The important role of COX in kidney development as well as kidney function early in life will be described in the proceeding paragraphs from: (a) reports of kidney dysfunction in children of pregnant women treated with COXI, (b) examination of animals with targeted COX-1 and COX-2 gene disruption, (c) effects of COXI on kidney development including nephrogenesis, and (d) effects of COXI on fetal and/or newborn kidney function in animal experiments.

### 3.1. COXI During Pregnancy: Effects on Fetal and Newborn Renal Function

Since the early 1970s, the non-selective COXI, indomethacin has been administered to pregnant women to inhibit the onset of premature labour [26]. Indomethacin readily crosses the placenta and the ratio of maternal to fetal serum indomethacin concentration in the human pregnancy is as high as 0.97 [27] resulting in equivalent drug concentrations in the mother and fetus regardless of gestational age. It is generally well recognized that exposure to NSAIDs may lead to hypoperfusion of the developing kidney as well as renal dysfunction in the newborn period. In fact, numerous studies have provided evidence of direct effects of indomethacin on kidney function in the fetus and newborn as described in the proceeding paragraphs.

In pregnant women treated with indomethacin tocolysis for 72 h, Kirshon *et al.* noted a decrease in fetal urine production [28]. In a population-based retrospective study of infants admitted into a neonatal intensive care unit during a five year period, 37 infants whose mothers received indomethacin for tocolysis were more likely than matched controls to have renal insufficiency (24% *vs.* 5%) [29]. The 10 day old kidneys of a twin delivered at 27 weeks after prenatal treatment with indomethacin from 24 weeks gestation showed a discontinuous and thin nephrogenic zone in the superficial cortex. The glomeruli and microtubules in the outer cortex were microcytic, there were reduced numbers of tubules in the cortical labyrinths and abnormalities in, and a paucity of, proximal convoluted tubules [30]. Taken together, these studies suggest that indomethacin treatment in the long-term (*i.e.*, >48 h), leads to alterations in both kidney development and kidney function [31,32,33].

The superiority of specific COX-2 over non-specific COXI in being devoid of adverse renal effects has been the subject of a randomized, double-blind, placebo controlled trial [34]. This study was designed to assess the long term use of the selective COX-2 inhibitor, rofecoxib, in human pregnancies at high risk of preterm delivery over a three year period, from 2,000 to 2,003 [35]. A reduction in fetal urinary production as well as amniotic fluid index was reported [34]; these effects on the fetal kidney were reversible, and ended after cessation of treatment. It is important to note, however, that rofecoxib did not reduce the incidence of early preterm labour (<30 weeks gestation) and was, in fact, associated with an increased incidence of delivery preterm (<37 weeks) in women at high risk [34].

In experiments in rabbits and sheep, the elimination of indomethacin from fetal plasma was found to be prolonged compared to that of adults [36,37], providing evidence that it can easily accumulate in fetal tissues. A number of investigations in animals have also provided evidence regarding the effects of maternal and/or fetal indomethacin administration on kidney function as follows: Stevenson and Lumbers measured the effects of acute I.V. injection of indomethacin to the pregnant ewe (10 mg/kg) and her fetus (12 mg/kg) on fetal renal function [38]. Using radiolabeled microspheres, renal blood flow to fetal sheep decreased minimally (<10%), whereas there was a significant reduction in cortical blood flow and a similar reduction in the ratio of inner to outer cortical blood flow as compared to control animals. These blood flow assessments were made ~2 h after indomethacin administration [38]. There were no significant effects on renal plasma flow, glomerular filtration rate or filtration fraction in fetal sheep, though there was a significant decrease in proximal tubular Na^+^ reabsorption of the fetal kidney as assessed using lithium clearance after ~1 h. There was also an increase in fetal urinary osmolality and a decrease in free water clearance by the fetal kidney from 1.5 to 3 h after indomethacin providing evidence that a single acute exposure of the fetal kidney to indomethacin impacts kidney function as well as intrarenal haemodynamics [38]. A single dose of indomethacin had no effect, however, on fetal urinary production, at least in the near-term healthy fetal sheep [38]. These observations confirmed the earlier report from Matson *et al.* [39] in which indomethacin (5 mg/kg bolus) was administered I.V. directly to the chronically instrumented fetal sheep. Glomerular perfusion decreased only in cortical regions of the fetal kidney and total glomerular filtration rate and urinary production remained unchanged after acute administration of indomethacin [39]. In addition, there was a decrease in plasma renin activity in the fetus demonstrating a modulatory role for endogenous PGs in regulating the renin-angiotensin system early in life [39]. Rac *et al.* measured the effects of a COX-2 inhibitor, meloxicam, administered to pregnant ewes in which premature labour was experimentally produced [40]. It was reported that 48 h of administration of meloxicam effectively inhibited the onset of premature labour, yet had no detrimental effects on either fetal renal blood flow or on fetal urinary production [40]. However, it is important to note that meloxicam also inhibits COX-1 [35] and is not highly selective for COX-2, as compared to such selective COX-2 inhibitors as refoxicob.

Studies by Walker *et al.* also evaluated the effects of indomethacin on renal function in conscious, chronically instrumented fetal sheep [41,42]. Indomethacin was infused I.V. over 5 h to the fetus (0.05 mg/kg bolus and 0.0025 mg/kg/min infusion) and measurements of renal function made at 60 min intervals. During this long-term infusion of indomethacin, fetal urinary flow rate decreased by ~50% after 1 h and remained low for the duration of the study whereas urinary osmolality increased after 3 h. There was also a decrease in free water clearance by ~70% which appeared to coincide with increased plasma levels of arginine vasopressin in the fetus [41]. In a follow-up study in fetal sheep, an arginine vasopressin V_2_ receptor antagonist was administered concomitantly with indomethacin. In these experiments, the decrease in fetal urinary flow rate following indomethacin was reversed and free water clearance increased in the presence of the V_2_ receptor antagonist [42]. These data suggest that the decrease in urinary production following prolonged exposure of the fetus to NSAIDs (such as occurs in the clinical setting and described below), may result from increased levels of arginine vasopressin.

### 3.2. COXI in the Newborn Period

Effects of COXI on renal development in rats and mice have focused on the role of COX-2. For example, in rats, the COX-2 inhibitor, rofecoxib was administered to dams from embryonic day 16 until delivery, and then to newborn pups for the first three weeks of postnatal life [43], the period of nephronogenesis. This treatment was associated with a reduction in nephron endowment as assessed by a decrease in glomerular number as well as glomerule volume [43]. There was also an elevation in systolic arterial pressure in the newborn period following perinatal treatment with rofecoxib as compared with vehicle [43]. These data support the findings from Komhoff *et al.* in which a COX-2 inhibitor was administered during pregnancy until weaning in the rodent, which significantly impaired development of the renal cortex and reduced glomerular diameter in both mice and rats [44]. Effects in rodents appear limited to the post natal phase of kidney development as detailed below.

### 3.3. Kidney Effects of Targeted Gene Disruption for COX

The development of COX deficient mice has allowed investigation into physiological effects of the two isoforms. The phenotypes of the two prostaglandin synthase genes (*Ptgs*, coding for COX-1 and COX-2) knockouts have revealed that the deficiency of COX-2 appears to have more profound developmental effects than deficiency with COX-1 [45]. Mice with a targeted gene disruption of *ptgs1*, which encodes COX-1 appeared normal and healthy. Pathological examination of the kidneys was remarkable only for the presence of a few small foci of basophilic, immature tubules per section examined; this remained unchanged up to five months of age [46]. This suggests that the COX-1 isoform is not required for normal kidney development. Interestingly, these mice exhibited a low-normal blood pressure when fed a normal salt diet (0.4%) but a low blood pressure when fed a low salt diet (<0.02%) or after treatment with an angiotensin converting enzyme inhibitor [47]. The mice also had a non-dipping pattern to their blood pressures measured during sleep possibly from enhanced sympathetic nervous system activity [48].

Conversely, mice with a targeted gene disruption of *ptgs2*, which encodes COX-2 have severe nephropathy evidenced as small, pale kidneys at eight weeks of age with a granular appearance of the capsule [49]. Microscopically, the lesions consist of multifocal areas of small immature glomeruli and tubules under the capsule with enlarged glomeruli outside of this hypoplastic area. By eight weeks of age, the kidneys also had scattered foci of tubular atrophy and interstitial fibrosis. By 16 weeks, the kidneys showed severe interstitial fibrosis, tubular atrophy and glomerular sclerosis. These changes were more pronounced in the homozygous male animals. The kidneys at day three were histologically normal, suggesting that COX-2 deficient mice had normal prenatal kidney development but demonstrated progressive changes within their kidneys following birth [49]. Adult null animals had corticomedullary microcysts and mild medullary hypoplasia accompanied by chronic kidney failure (defined as a 50% reduction in GFR) [50] and in some cases, the animals died from peritonitis [51]. The blood pressures of these COX-2 deficient animals were normal, as were fluid intakes, urinary outputs, urinary osmolalities, and urinary electrolytes [50] but this may depend somewhat on the background strain of the mice studied [52]. The average lifespan of the null mice was 3.5 months but animals occasionally survived to >6 months of age. The aforementioned renal changes are also exhibited in animals with only a partial disruption of COX-2 expression, though to a lesser extent: Seta *et al.* created hypomorphic Ptgs (COX-2(Neo/Neo)) mice in which COX-2 expression was suppressed but not completely eliminated. These mice developed a mild renal phenotype with minimal signs of renal dysfunction [53]. Taken together, then, it appears that the COX-2 isoform plays a predominant role in kidney development, likely through the production of endogenous PGs.

### 3.4. COXI during Postnatal Nephronogenesis

It is well known that administration of NSAIDs to preterm infants with symptomatic *patent ductus arteriosus* is associated with significant renal impairment. In order to investigate whether ibuprofen has less adverse effects than indomethacin, newborn rats received intraperitoneal injections of either NSAID on the first three days postnatally [54]. With indomethacin treatment (0.2 mg/kg on postnatal day 1, then 0.1 mg/kg on postnatal days 2 and 3), PGE_2_ levels and COX-2 mRNA expression were decreased in whole kidney homogenates while levels of PGF_2__α_ were increased [54]. In contrast, ibuprofen treatment (10 mg/kg on postnatal day 1, then 5 mg/kg on days 2 and 3) increased COX-2 mRNA levels [54] although this medication has less COX-2 selectivity than indomethacin [55,56].

In experiments conducted in newborn mice, either selective COX-2 inhibitors (SC-236, celecoxib, rofecoxib, etoricoxib, valdecoxib or lumiracoxib), a selective COX-1 inhibitor (SC-560) or non-selective COX inhibitors (diclofenac or naproxen), were injected subcutaneously from postnatal days one to six and intraperitoneally from days 7 to 21 [57]. COX-1 inhibitors had no effect on kidney morphology while the COX-2 inhibitors and the non-selective NSAIDs all affected nephrogenesis, but to differing degrees. SC-236 affected kidney growth, glomerular size and cortical area to a milder extent than seen in COX-2 knockout mice [57]. The non-selective COX inhibitors appeared to have the greatest effect on kidney development (attenuation of both glomerular and subcapsullar growth). In contrast to that seen in COX-2^−/−^ mice, juxtamedullary glomeruli were also adversely affected by both the NSAIDs and COX-2 inhibitors. Olliges *et al.* concluded that the classical NSAIDs, declofenac and naproxen, caused the most marked defects in renal development among all the inhibitors tested in this study [57].

Kent *et al.* evaluated the effects of either ibuprofen or indomethacin on nephron endowment in the Sprague Dawley rat pup. In this study, drugs were administered intraperitoneally for the first 5 postnatal days in rat pups and evaluations of glomerular number made at day 14 of postnatal life. There was no reduction in total glomerular number assessed using the physical dissector/fractionator combination method for stereology following treatment with these non-selective COXI [58]. In another study, Saez *et al.* showed that administration of the selective COX-2 inhibitor, rofecoxib, from embryonic day 16 to postnatal day 21 resulted in a significant but modest reduction in glomerular number measured in adult rats, as determined using the same stereological method [43]. Taken together, these studies again provide evidence that it is the COX-2 isoform which appears to have the most impact on renal development.

### 3.5. Physiological Effects of COXI on the Newborn Kidney

Administration of a COXI has been the preferred treatment for infants with symptomatic patent *ductus arteriosus* (PDA) [59] for several decades. Effective closure of a PDA reduces the need for surgical ligation as well as the incidence of intraventricular cerebral hemorrhage, necrotising encolitis, bronchopulmonary dysplasia, and death, and is therefore considered an important therapeutic regimen. Currently, ibuprofen appears to be the therapeutic option of choice in the preterm human infant with PDA because of its better renal tolerability compared to other NSAIDs, such as the more traditionally administered indomethacin [60]. Nevertheless, the use of ibuprofen is not free from adverse renal effects, particularly in circumstances when renal PG activation is maximal and there are numerous side effects associated with NSAID treatment including oliguria, which if untreated can result in AKI in the neonatal period [61]. This suggests that PG’s may play a vital role in promoting perfusion of the newborn kidney. 

Pezzati *et al.* [62] showed that in mechanically ventilated preterm infants, there was a significant decrease in renal blood flow velocity measured using Doppler ultrasound within 30 min of receiving the NSAIDs indomethacin or ibuprofen. It is important to note that kidney development may still proceed in human infants born prematurely [63]. Therefore, NSAID treatment of preterm infants could also impact renal development, including nephron endowment. In fact, the effect of postnatal treatment of these infants with COXI has not fully been elucidated but may be difficult to determine in the absence of other renal insults. In a large (n = 148) controlled study evaluating the effects of ibuprofen on kidney function in premature infants, Vieux *et al.* reported that treated infants had a lower glomerular filtration rate for the first month of life and exhibited a higher fractional excretion of Na^+^ and urinary albumin:creatinine ratio as compared with infants who received no ibuprofen [64] confirming earlier reports [33,65] and implicating NSAIDS as nephrotoxic to the premature kidney. In addition, coadministration of NSAIDs with aminoglycosides, or vancomycin, could be more nephrotoxic than NSAIDs alone (see also Antonucci *et al.* [31]). Therefore, isolating the physiological effects of NSAIDs alone as well as the role of endogenously produced PGs is the first step in understanding their impact on other drugs or other pathways which could also potentially impact kidney development and kidney function. Our understanding of the role of endogenously produced PGs in influencing the function of the newborn kidney is described in the proceeding paragraphs from experiments conducted in rabbits, piglets, and sheep.

PGs are biosynthesized early in kidney development perhaps providing important physiological roles in modulating kidney function during fetal and newborn life [66]. Previous studies in experimental animals have investigated the role of PG’s produced by the fetal and newborn kidney in modulating renal haemodynamics, yet the results are variable. This variability may reflect differences in experimental design, state of the animal, method of measurement of renal haemodynamics, species, choice of drug, and dose: For example, Herin & Aperia [67] showed in anaesthetised, paralysed lambs that indomethacin (2.5 mg/kg bolus plus 1.0 mg/h/kg) had no effect on renal blood flow measured using radio-labelled microspheres. In conscious lambs, Winther *et al.* [68] reported a decrease in effective renal blood flow, and calculated from the clearance of radio-labelled hippurate at two to four hours after administration of low dose (0.2 mg/kg) as well as high dose (7.5 mg/kg) indomethacin. They also showed a further decrease at 12–14 h and again at 22–24 h after administration of high but not low dose indomethacin. In conscious older piglets but not newborn piglets, Osborn *et al.* [69] showed that indomethacin (bolus 3.0 mg/kg plus infusion of 2.0 mg/h/kg) decreased kidney blood flow and increased the ratio of outer:inner cortical flow, as measured with radiolabelled microspheres. Studies in anaesthetised and ventilated newborn rabbits [70,71] have provided evidence that administration of other COX inhibitors-ibuprofen and acetylsalicyclic acid-increase renal vascular resistance within 30 to 60 min. Comparative effects of I.V. aspirin, indomethacin, and ibuoprofen were also measured in anaesthetised, ventilated newborn rabbits by the same group [61]. All COXI exhibited marked effects in increasing renal vascular resistance within 30 to 60 min. In these experiments, renal vascular resistance was calculated from the measurement of effective renal plasma flow as the clearance of para-aminohippurate. 

Less is understood regarding the function of the newborn kidney and how NSAIDs might impact glomerular or tubular function. For example, Prevot *et al.* [72] measured the effects of the COX-2 inhibitor, nimesulide, in pentobarbital anesthetized newborn rabbits. A decrease in urinary flow rate, glomerular filtration rate and renal blood flow were reported, with the most dramatic effects being observed at the highest doses administered (200 mg/kg bolus plus 5 mg/kg/min infused I.V. for 60 min). In these studies in which experiments were carried out immediately after surgery and in the presence of anaesthesia, it is important to note that both anaesthesia and surgery can impact directly upon the measured variables and therefore, the experimental results. More recently, we showed that endogenously produced PGs are also potent renal vasodilators in the newborn period, as evidenced by a dramatic renal vasoconstriction following administration of indomethacin to conscious lambs [73]: In our experiments carried out in conscious, chronically instrumented lambs, we measured the cardiovascular effects of indomethacin at two postnatal ages resulting in the following novel findings: Following administration of indomethacin but not vehicle in both age groups, there was an increase in arterial pressure and pulse interval and a marked increase in renal vascular resistance. These effects of indomethacin were, however, transient with baseline levels being reached within minutes. These findings show that under physiological conditions, endogenously produced PGs modulate both systemic and renal haemodynamics early in life. Other vasoactive factors must, however, be rapidly recruited which serve to buffer the circulatory responses to the removal of vasodilatory PGs. Therefore, any sustained effects of indomethacin on renal perfusion which could impact renal function leading to oliguria if untreated, could only occur in the absence of the rapid buffering capacity we observed in the healthy newborn animal. That is, if there was a lack of release of vasodilatory factors such as nitric oxide (NO) and/or an increased release of vasoconstrictor factors such as angiotensin II (ANG II), the effects of NSAIDs on renal vascular resistance could be sustained leading to more deleterious consequences on the function of the newborn kidney.

In support of this notion, in our previous studies in conscious lambs we showed a greater modulatory role for NO on the renal circulation early in life [74,75,76]. In other experiments, the role of endogenously produced PGs in regulating the renal haemodynamic responses to the potent vasoconstrictor, endothelin-1 (ET-1), was studied in two age groups of conscious lambs (~one and ~six weeks). In this study, renal haemodynamic effects of ET-1 were measured before and after intra-arterial injection of ET-1 both before and after pre-treatment with either vehicle or indomethacin (1 mg/kg) in two age groups of conscious lambs. Renal haemodynamic responses to ET-1 were not altered by pretreatment with indomethacin at either postnatal age. These results suggest that endogenously produced PGs do not modulate the renal haemodynamic effects of ET-1 in conscious lambs during postnatal maturation [77]. In contrast, more recently, we measured the pressor and renal blood flow responses to ANG II before and after administration of the L-arginine analogue, *N*^G^-nitro-L-arginine methyl ester (L-NAME) which prevents the production of NO, as well as after treatment with either vehicle, or indomethacin (1 mg/kg) [78]. In both age groups, the pressor and renal vasoconstrictor responses to ANG II were augmented by pretreatment with indomethacin, the effects being similar at one and six weeks. The haemodynamic responses to ANG II were, however, not altered after L-NAME following pretreatment with either vehicle or indomethacin. These data provide new evidence that soon after birth, endogenously produced PGs, but not endogenously produced NO balance the vasoconstrictor actions of ANG II. There is, however, no apparent interaction between PGs and NO in modulating the responses to ANG II postnatally [78].

Together then, results from experiments in conscious newborn animals detailed above support the notion that administration of COXI could potentially have deleterious effects on renal haemodynamics in the developing newborn which supports the clinical evidence. We speculate that if the marked effects of COXI on renal vascular resistance such as we observed with acute indomethacin administration to conscious lambs, persist in the absence of the rapid buffering capacity observed in the healthy newborn animal, the perfusion of the kidney would decrease, resulting in a decreased capacity to filter the blood, and leading eventually to acute kidney injury, and death if the kidney perfusion is not restored.

## 4. Summary

The finding of altered kidney development in the offspring of mothers treated with COXI suggests that a reduction in PG levels during fetal life may affect nephrogenesis. The reduction in COX-2 expression (knockout animals) or activity (COXI) after birth results in alterations only in postnatal kidney development which might suggest that maternal PGs or those produced in the fetus by COX-1 might be important for fetal kidney development including nephrogenesis. In addition, there appears to be an absolute requirement for COX-2 in achieving a normal renal phenotype after birth. Our physiological findings support the notion that COXI in the short-term have no deleterious effects on kidney resistance since it appears that vasodilatory factors are rapidly released in order to buffer the constrictor responses to COXI under physiological conditions. However, if release of vasodilatory factors was prevented, a sustained increase in renal vascular resistance and subsequently, a decrease in perfusion and filtration would ensue. Our studies to date demonstrate that the balance of vasoconstrictor to vasodilatory factors is the key underlying component to sustained kidney perfusion in the immediate post natal period. Less is understood regarding the impact of COXI on glomerular and tubular function of the newborn kidney, though the clinical evidence points to a marked impact of COXI on both. More experimental studies evaluating the effects of non-selective and selective inhibitors of COX isoforms on renal function in the developing newborn are clearly warranted. 

## 5. Conclusions

This review has provided evidence that COXI either during pregnancy or after birth can influence kidney development including nephronogenesis, and can decrease renal perfusion and ultrafiltration potentially leading to acute kidney injury in the newborn period. To date, which COX isoform plays a more important role in during fetal development and influences kidney function early in life is not known, though evidence points to a predominant role for COX-2. Caution should, however be taken when administering any COXI long-term during the perinatal period due to the known deleterious effects on newborn kidney function. Moreover, one should note that in addition to the duration of administration of COXI, the dose also needs to be carefully considered. While it is generally agreed that the development of a completely renal-sparing COXI is unlikely, the role of COXI in treating premature labour, as well as PDA in the preterm infant should be carefully considered especially in light of the dose and duration of administration of COXI. Importantly, due to the evidence accumulating of a direct impact of NSAIDs on glomerular ultrafiltration in the preterm infant, any drugs that are eliminated by filtration during this time should be handled cautiously and kidney function carefully monitored.
